# Inhibition of FcRn with rozanolixizumab in adults with immune thrombocytopenia: Two randomised, double‐blind, placebo‐controlled phase 3 studies and their open‐label extension

**DOI:** 10.1111/bjh.19858

**Published:** 2024-11-18

**Authors:** Nichola Cooper, James B. Bussel, Maciej Kaźmierczak, Yoshitaka Miyakawa, Sarah Cluck, Rocío Lledó García, Birgit Haier, Andreea Lavrov, Puneet Singh, Rose Snipes, David J. Kuter

**Affiliations:** ^1^ Imperial College London London UK; ^2^ Weill Cornell Medicine – New York Presbyterian Hospital New York New York USA; ^3^ Poznań University of Medical Sciences Poznań Poland; ^4^ Saitama Medical University Saitama Japan; ^5^ UCB Slough UK; ^6^ UCB Monheim Germany; ^7^ UCB Morrisville North Carolina USA; ^8^ Hematology Division Massachusetts General Hospital Boston Massachusetts USA

**Keywords:** clinical trial, FcRn, immune thrombocytopenia, immunoglobulin G, open‐label extension, rozanolixizumab

## Abstract

Primary immune thrombocytopenia (ITP) is an antiplatelet‐antibody‐mediated disorder with accelerated platelet clearance and decreased platelet production. Rozanolixizumab, a monoclonal IgG4 anti‐FcRn antibody, blocks IgG recycling and decreases IgG levels. We report efficacy and safety of rozanolixizumab in adults with persistent/chronic ITP in 24‐week phase 3 studies (TP0003; TP0006), and their 52‐week open‐label extension (OLE). Primary end‐point was durable clinically meaningful platelet response (DCMPR) of ≥50 × 10^9^/L for 8/12 weeks during Weeks 13–25 in the double‐blind studies. Operational delays and evolving ITP treatment landscape led the sponsor to terminate these studies early; thus, only 21 and 12 (TP0003) and 20 and 10 (TP0006) patients were randomised to rozanolixizumab or placebo. Forty‐three patients enrolled in the OLE: 42 started on every 2‐week dosing; 21 later switched to weekly dosing. More rozanolixizumab‐treated than placebo‐treated patients achieved DCMPR: 4/21 versus 0 (TP0003) and 1/20 versus 0 (TP0006). Platelet increases to ≥50 × 10^9^/L were observed on Day 8 in 52.4% (TP0003; 2/12 placebo) and 45.0% (TP0006; 1/10 placebo) of rozanolixizumab‐treated patients. OLE platelet increases were maintained while on weekly dosing. The most frequent treatment‐emergent adverse events overall were headache, pyrexia and nausea, as seen previously. Weekly dosing appears more efficacious than every 2‐week dosing.

## INTRODUCTION

Primary immune thrombocytopenia (ITP) is an antiplatelet‐antibody‐mediated disorder characterised by accelerated platelet clearance and decreased platelet production, resulting in variable bleeding and bruising.^1^, ^2^, ^3^, ^4^ Current management of ITP includes first‐line treatment with glucocorticoids and sometimes intravenous immunoglobulin, and second‐line treatment with anti‐CD20 antibodies, thrombopoietin‐receptor agonists, spleen tyrosine kinase inhibitors, other immunosuppressants and splenectomy,^5^ with the goal of sustaining adequate platelet counts and preventing bleeding.^5^, ^6^ Responses to treatment may initially be successful; however, even successfully treated patients usually require ongoing therapy which may be associated with diminished efficacy and/or side effects.^5^ Thus, new therapeutic strategies aim to optimise efficacy, limit side effects and improve health‐related quality of life.^5^

FcRn recycles serum IgG and albumin, which prolongs their half‐lives by preventing their lysosomal degradation.^7^ One such therapeutic strategy is inhibition of the neonatal Fc receptor (FcRn) which reduces IgG levels, including antiplatelet IgG autoantibodies. Rozanolixizumab is a humanised monoclonal IgG4 antibody that targets the IgG‐binding region of FcRn, and lowers IgG levels by inhibiting IgG recycling.^7^ Because rozanolixizumab binds FcRn distinct from the albumin binding site, clinically meaningful reductions in albumin levels have not been observed.^8^, ^9^, ^10^ In a phase 2 study (NCT02718716) of rozanolixizumab in 66 patients with persistent/chronic primary ITP, rozanolixizumab demonstrated an acceptable safety profile; rapid, substantial platelet increases and marked IgG reductions at the higher doses, with no clinically meaningful changes in other immunoglobulins, albumin or infections.^9^ We report efficacy, safety and tolerability of rozanolixizumab in adults with persistent/chronic ITP from twin double‐blind, placebo‐controlled phase 3 studies and their open‐label extension (OLE).

## METHODS

### Double‐blind studies

#### Study design and participants

TP0003 (NCT04200456) and TP0006 (NCT04224688) were identical double‐blind, randomised, placebo‐controlled phase 3 studies in patients aged ≥18 years with a diagnosis of persistent (3‐ to 12‐month duration^11^) or chronic (>12‐month duration^11^) primary ITP. Patients were recruited in North America, Europe and Asia from 29 sites for TP0003 and a separate 29 sites for TP0006.

Eligible patients in both studies had platelets <30 × 10^9^/L at screening and baseline, intolerance or insufficient response to ≥2 standard‐of‐care ITP treatments prior to screening, and history of response to a previous ITP therapy. Concomitant oral corticosteroids, mycophenolate mofetil, ciclosporin, azathioprine, danazol, dapsone, eltrombopag, avatrombopag and fostamatinib at a stable dose for up to 2 months prior to baseline were permitted. Any increase in concomitant ITP medication dose was considered rescue therapy. Patients were excluded if they had thromboembolism within 6 months prior to randomisation, bleeding warranting platelet transfusion, known hypersensitivity to the study medication, clinically relevant active or recent serious infection, or if they were pregnant or breastfeeding.

The sample size was determined based on the observed durable response rates from a previous randomised controlled trial of romiplostim in patients with chronic ITP.^12^ Using the true response rate of 0.45 vs. 0.05 from Kuter et al.,^12^ a sample size of 60 participants randomised in a 2:1 ratio would provide >90% power to detect a statistically significant difference between treatment groups using Fisher's exact test at an alpha level of 0.025 (one‐sided).

#### Interventions

Patients were randomised 2:1 to receive rozanolixizumab or matched placebo using interactive response technology. Randomisation was stratified by platelet count < or ≥15 × 10^9^/L, and history of splenectomy (yes or no). These stratification factors were selected to ensure consistency of effect across disease severity and prior treatments. Because of the small number of patients anticipated at individual sites, randomisation was done across, rather than within, sites. Site staff were masked to treatment allocation, while site pharmacists had access.

A fixed‐unit dosing regimen across body weight tiers equivalent to (≈) 15 mg/kg (starting dose) followed by ≈10 mg/kg (maintenance doses) every 2 weeks was decided for use in TP0003 and TP0006 based upon the platelet response (>50 × 10^9^/L), the time to onset of the platelet count increase, and the safety profile observed in the phase 2 study, in which a single dose of 15 mg/kg reached a 52% decrease in IgG from baseline by Day 8.^9^ The study also showed that the median durations of platelet response following a dose of 2 × 10 mg/kg weekly doses or a single dose of 15 mg/kg were both 12 days.^9^ Hence, a starting dose of ≈15 mg/kg followed by ≈10 mg/kg every 2 weeks was expected to sustain a reduction from baseline in IgG levels by >50% and to be sufficient to translate into a relevant platelet response.

A screening period of 4 weeks was followed by a 24‐week treatment period. On Day 1, patients received a subcutaneous (SC) infusion of rozanolixizumab ≈15 mg/kg or placebo (Figure S1A). Patients subsequently received ≈10 mg/kg infusions of rozanolixizumab or placebo, every 2 weeks. Down‐titration was permitted if required to ≈7 mg/kg or, in a second step, to a 280 mg total dose every 2 weeks, based on platelet count and adverse events (Table S1). Up‐titration after down‐titration was also possible. After Week 25, patients were followed for six more weeks.

#### Outcomes

Clinical response criteria were the same for both studies. The primary efficacy end‐point was a durable clinically meaningful platelet response (DCMPR): ≥50 × 10^9^/L for ≥8/12 visits during Weeks 13–25. Secondary efficacy end‐points included: cumulative weeks with platelets ≥50 × 10^9^/L, time to first platelet response ≥50 × 10^9^/L, response by Day 8 and number of patients with ≥2 platelet counts of ≥30 × 10^9^/L and a ≥ 2‐fold increase from baseline with absence of bleeding. Safety assessments included incidence of treatment‐emergent adverse events (TEAEs), TEAEs leading to permanent withdrawal of treatment, serious TEAEs, treatment‐related TEAEs, adverse events of special monitoring (AESMs; severe headache, diarrhoea, abdominal pain, vomiting, opportunistic infections, and arterial/venous thromboembolic events) and changes from baseline in vital signs, electrocardiograms and laboratory values. TEAEs were classified according to the Common Terminology Criteria for Adverse Events (CTCAE) for severity, or given a standard intensity classification (mild, moderate or severe) if CTCAE grading was not possible. All serious TEAEs and non‐serious AESMs were followed until resolution, stabilisation, the investigator determined that it was no longer clinically significant, the event was otherwise explained, or the participant was lost to follow‐up. Pharmacokinetic (PK) and pharmacodynamic outcomes were rozanolixizumab concentrations and serum IgG levels. Incidence and emergence of anti‐drug antibodies (ADAs) were also assessed.

### Open‐label extension

#### Study design and participants

Patients who completed the 24‐week double‐blind treatment period could opt to enrol into TP0004 (NCT04596995), a 1‐year OLE study. Patients were excluded if they had ongoing serious adverse events or study‐drug‐related severe TEAEs, had a change in medical condition such that they no longer met the initial inclusion criteria or had significant bleeding requiring immediate treatment.

#### Intervention

To maintain blinding, patients started the OLE at the same rozanolixizumab dose if the platelet count was ≥50 × 10^9^/L and ≤150 × 10^9^/L. Up‐ and down‐titrations were permitted (Figure S1B).

Patients initially received rozanolixizumab every 2 weeks; however, based upon a recommendation from the Independent Data Monitoring Committee for an alternative dosing regimen, subsequent protocol amendments required patients to switch to a weekly dosing regimen (Figure S1B). Patients enrolling into the OLE after local approval of the protocol amendment received weekly infusions (no patients enrolled in TP0003 and TP0006 after the amendments). Patients were able to receive rescue therapy at the investigator's discretion, but this could trigger withdrawal from the study depending on the type and dose of rescue therapy administered. There was an 8‐week follow‐up period after the last dose.

#### Outcomes

Primary outcomes were incidence of TEAEs and TEAEs leading to treatment withdrawal. Safety was additionally assessed by the incidence of AESMs, serious TEAEs, treatment‐related TEAEs and changes from baseline in vital signs, electrocardiogram and laboratory values. Stable clinically meaningful response (mean platelets ≥50 × 10^9^/L), mean change from baseline in platelet count at each visit, rozanolixizumab concentration, serum IgG levels and incidence and emergence of ADAs were also assessed. The occurrence of ADA‐positive samples detected at ≥2 consecutive sampling timepoints during treatment, where the first and last ADA‐positive samples were separated by ≥16 weeks, was defined as persistent ADA.

### Study termination

Due to the evolving ITP treatment landscape with multiple new targeted therapies being offered or in late‐stage clinical development, the study sponsor chose to terminate these studies. Since study termination was not triggered by safety concerns, ongoing study participants could complete the double‐blind studies and continue into the OLE. However, early study termination prevented the achievement of planned sample size, and amendments to the protocols to allow weekly dosing in the double‐blind studies were not implemented as the study was terminated before these came into effect.

### Statistical analyses

Due to early termination, the planned sample size for the double‐blind studies was not reached, and efficacy analyses were reported descriptively. Safety analyses were prespecified as descriptive.

Efficacy analyses for the double‐blind studies included all randomised patients (Randomised Set). Safety analyses included randomised patients who received ≥1 dose of rozanolixizumab (Safety Set). For the OLE, efficacy and safety analyses were both performed using the Safety Set. Patients who discontinued treatment due to TEAEs or rescue therapy were considered non‐responders. PK analyses included patients who received ≥1 dose of rozanolixizumab and had ≥1 valid PK measurement. SAS version 9.2 or later was used for data processing.

## RESULTS

### Study population

From 31/01/2020 to 06/04/2022 (TP0003), and 03/06/2020 to 11/11/2021 (TP0006), 157 patients were screened for the double‐blind studies. In total, 70 patients (28 from TP0003; 42 from TP0006) were screening failures due to ineligibility (Figure S2). Enrolled patients were randomised 2:1 to receive rozanolixizumab or placebo in TP0003 (*n* = 33) and TP0006 (*n* = 30), with 43/63 patients across both studies enrolling into the OLE. Five/twenty‐one and 2/20 patients in TP0003 and TP0006, down‐titrated from ≈10 to ≈7 mg/kg; no patient reduced to 280 mg. All patients started the OLE (TP0004) on every‐2‐week dosing, except one. Twenty‐one of 42 patients switched to weekly dosing after implementation of the protocol amendment.

Baseline demographics and disease characteristics were generally balanced between treatment groups and across studies (Table 1). Most patients had chronic ITP; rozanolixizumab‐treated patients with chronic ITP enrolled in TP0006 had longer median disease duration (12.1 years) than those enrolled in TP0003 (5.2 years). More patients in TP0006 (56.7%) had used ≥3 prior ITP medications compared to TP0003 (36.4%).

**TABLE 1:** Patient baseline demographics and disease characteristics.

	TP0003 (double‐blind)			TP0006 (double‐blind)			TP0004 (OLE)
	RLZ	Placebo	All patients	RLZ	Placebo	All patients	RLZ
	*n* = 21	*n* = 12	*N* = 33	*n* = 20	*n* = 10	*N* = 30	*N* = 43
Age, years, median (range)^a^	39.0 (18, 71)	50.5 (22, 78)	42.0 (18, 78)	41.0 (18, 72)	41.5 (20, 60)	41.0 (18, 72)	42.0 (18, 78)
Gender, female, *n* (%)	12 (57.1)	11 (91.7)	23 (69.7)	13 (65.0)	6 (60.0)	19 (63.3)	28 (65.1)
Weight, kg, median (range)	74.0 (52.0, 118.0)	71.0 (57.9, 122.0)	73.0 (52.0, 122.0)	72.5 (51.9, 115.0)	67.6 (52.0, 95.0)	71.1 (51.9, 115.0)	73.3 (54.6, 121.6)
Body weight category, *n* (%)							
50–<70 kg	7 (33.3)	6 (50.0)	13 (39.4)	8 (40.0)	6 (60.0)	14 (46.7)	18 (41.9)
70–<100 kg	11 (52.4)	4 (33.3)	15 (45.5)	7 (35.0)	4 (40.0)	11 (36.7)	19 (44.2)
≥100 kg	3 (14.3)	2 (16.7)	5 (15.2)	0	0	0	6 (14.0)
Race, *n* (%)							
Asian	4 (19.0)	1 (8.3)	5 (15.2)	6 (30.0)	3 (30.0)	9 (30.0)	8 (18.6)
White	16 (76.2)	11 (91.7)	27 (81.8)	13 (65.0)	7 (70.0)	20 (66.7)	33 (76.7)
Other or mixed	0	0	0	0	0	0	1 (2.3)
Missing	1 (4.8)	0	1 (3.0)	1 (5.0)	0	1 (3.3)	1 (2.3)
Region, *n* (%)							
North America	0	0	0	1 (5.0)	0	1 (3.3)	1 (2.3)
Europe	17 (81.0)	11 (91.7)	28 (84.8)	13 (65.0)	7 (70.0)	20 (66.7)	34 (79.1)
Asia (excluding Japan)	1 (4.8)	1 (8.3)	2 (6.1)	6 (30.0)	3 (30.0)	9 (30.0)	7 (16.3)
Japan	3 (14.3)	0	3 (9.1)	0	0	0	1 (2.3)
Age at first ITP diagnosis, years, median (range)	33.0 (8, 66)	46.0 (6, 78)	39.0 (6, 78)	34.5 (2, 64)	29.0 (3, 59)	33.0 (2, 64)	38.0 (2, 78)
Time since first confirmed diagnosis of ITP, years, median (range)^b^	4.1 (0.3, 31.3)	8.7 (0.4, 31.6)	4.6 (0.3, 31.6)	7.2 (0.4, 26.4)	4.8 (0.6, 29.9)	6.1 (0.4, 29.9)	5.1 (0.9, 32.2)
Splenectomy							
Yes, *n* (%)	2 (9.5)	1 (8.3)	3 (9.1)	3 (15.0)	1 (10.0)	4 (13.3)	3 (7.0)
IgG level, g/L, median (range)	‐ (9.6, 15.8)	‐ (12.7, 12.7)	12.7 (9.6, 15.8)	12.1 (10.4, 15.4)	‐ (9.5, 9.5)	11.2 (9.5, 15.4)	8.7 (6.8, 11.8)
No, *n* (%)	19 (90.5)	11 (91.7)	30 (90.9)	17 (85.0)	9 (90.0)	26 (86.7)	40 (93.0)
IgG level, g/L, median (range)	9.8 (5.2, 17.6)	9.7 (5.8, 19.9)	9.7 (5.2, 19.9)	11.0 (6.9, 15.3)	8.7 (6.0, 11.5)	10.0 (6.0, 15.3)	6.0 (2.2, 18.9)
Platelet count (×10^9^/L), median (range)^b^	19.0 (1.0, 34.0)	17.5 (1.0, 31.0)	18.0 (1.0, 34.0)	16.0 (1.0, 26.0)	14.0 (3.0, 24.0)	16.0 (1.0, 26.0)	28.0 (1.0, 295.0)
Degree of thrombocytopenia: platelet count, *n* (%)							
≥15 × 10^9^/L	14 (66.7)	8 (66.7)	22 (66.7)	11 (55.0)	5 (50.0)	16 (53.3)	31 (72.1)
<15 × 10^9^/L	7 (33.3)	4 (33.3)	11 (33.3)	9 (45.0)	5 (50.0)	14 (46.7)	12 (27.9)
ITP category, *n* (%)							
Persistent ITP^c^	4 (19.0)	1 (8.3)	5 (15.2)	4 (20.0)	1 (10.0)	5 (16.7)	2 (4.7)
Chronic ITP^c^	17 (81.0)	11 (91.7)	28 (84.8)	16 (80.0)	9 (90.0)	25 (83.3)	41 (95.3)
ITP duration							
Persistent ITP, months, median (range)^c^	5.8 (3.7, 10.2)	‐ (4.2, 4.2)	5.1 (3.7, 10.2)	6.7 (4.3, 9.7)	‐ (7.4, 7.4)	7.4 (4.3, 9.7)	‐ (10.7, 11.3)
Chronic ITP, years, median (range)^c^	5.2 (1.0, 31.3)	8.9 (1.2, 31.6)	5.8 (1.0, 31.6)	12.1 (1.4, 26.4)	4.8 (1.3, 29.9)	12.1 (1.3, 29.9)	5.3 (1.1, 32.2)
Total number of prior ITP medications, median (range)	4.0 (2, 28)	3.0 (1, 45)	4.0 (1, 45)	6.5 (2, 34)	6.5 (3, 15)	6.5 (2, 34)	6.0 (1, 52)
Total number of prior unique^d^ ITP medications, median (range)	2.0 (1, 9)	2.0 (1, 5)	2.0 (1, 9)	2.5 (1, 12)	3.0 (2, 5)	3.0 (1, 12)	3.0 (1, 12)
Number of prior unique^d^ ITP medications, *n* (%)							
1	4 (19.0)	3 (25.0)	7 (21.2)	2 (10.0)	0	2 (6.7)	7 (16.3)
2	9 (42.9)	5 (41.7)	14 (42.4)	7 (35.0)	2 (20.0)	9 (30.0)	12 (27.9)
≥3	8 (38.1)	4 (33.3)	12 (36.4)	9 (45.0)	8 (80.0)	17 (56.7)	24 (55.8)
Missing	0	0	0	2 (10.0)	0	2 (6.7)	0
Baseline use of TPO‐RAs^e^							
Yes, *n* (%)	3 (14.3)	0	3 (9.1)	2 (10.0)	1 (10.0)	3 (10.0)	5 (11.6)
No, *n* (%)	18 (85.7)	12 (100)	30 (90.9)	18 (90.0)	9 (90.0)	27 (90.0)	38 (88.4)

*Note*: Median values for subgroups with one or two patients cannot be calculated and are marked with a ‘‐’.

Abbreviations: ATC, anatomical therapeutic chemical; eCRF, electronic case report form; IgG, immunoglobulin G; ITP, immune thrombocytopenia; OLE, open‐label extension; RLZ, rozanolixizumab; TPO‐RA, thrombopoietin‐receptor agonist.

^a^ Missing age is calculated using year of birth as detailed on Informed Consent Form.

^b^ As collected in the eCRF.

^c^ Persistent ITP is >3 months but <12 months, and chronic ITP is ≥12 months since diagnosis.

^d^ Unique medications were derived by counting all medications with ATC code H02 as one, all medications with ATC code J06 as one and remaining prior ITP medications by counting all unique standardised medication names (CMDECOD).

^e^ For example, eltrombopag and/or avatrombopag, but not romiplostim.

### Efficacy

**FIGURE 1:**
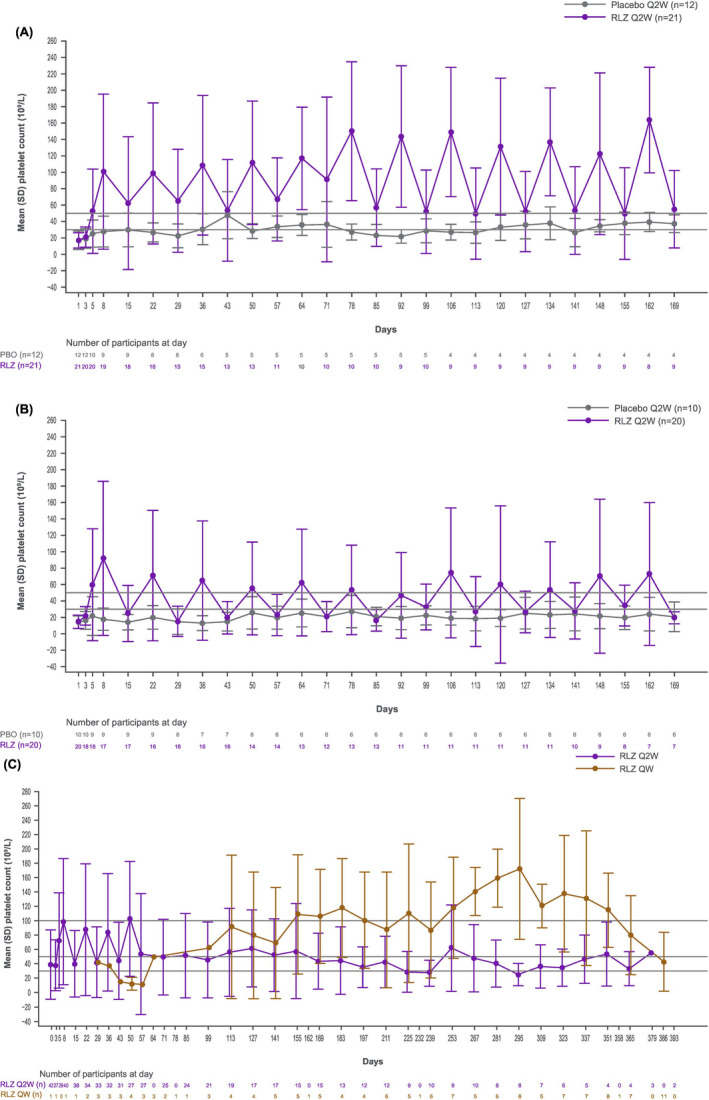
Mean platelet count over time. (A) TP0003 (double‐blind)*. (B) TP0006 (double‐blind)*. (C) TP0004 (open‐label extension)^†^. Excludes observations occurring after the administration of rescue medication. *Platelet counts were measured on Day 3, Day 5 and Day 8, and weekly thereafter. ^†^Until Day 57, platelet counts were measured every week. Thereafter, platelet counts were measured every 2 weeks (prior to dosing in the Q2W regimen). Q2W, every 2 weeks; QW, weekly; RLZ, rozanolixizumab; SD, standard deviation.

Increases in platelet counts (to ≥50 × 10^9^/L) were observed as early as Day 8 in 11/21 (TP0003) and 9/20 (TP0006) patients treated with rozanolixizumab at the initial ≈15 mg/kg dose, and in 2/12 (TP0003) and 1/10 (TP0006) of placebo controls. During TP0003, mean platelet counts of ≥50 × 10^9^/L were repeatedly observed 1 week postdose; however, the effect was not maintained over the full 2‐week dosing interval (Figure 1A,B); this degree of efficacy was seen less frequently in TP0006. In TP0003 and TP0006, 4/21 and 1/20 rozanolixizumab‐treated patients achieved a DCMPR; no placebo patients achieved this end‐point. Mean platelet counts of ≥50 × 10^9^/L (measured every second week after Day 57) were maintained in the OLE during weekly dosing but not every‐2‐week dosing (Figure 1C).

In TP0003, rozanolixizumab‐treated patients had a mean (SD) time with platelets ≥50 × 10^9^/L of 7.6 (9.0) weeks versus 1.3 (2.5) weeks for placebo‐treated patients. In TP0006, platelets ≥50 × 10^9^/L lasted 4.0 (5.4) weeks with rozanolixizumab treatment versus 1.4 (3.2) weeks with placebo. The Kaplan–Meier median time (time at which probability of patients achieving platelets ≥50 × 10^9^/L is 50%) was 8 days for both rozanolixizumab groups and 44 days in TP0003 for placebo (it could not be calculated for the placebo group in TP0006). A greater proportion of patients receiving rozanolixizumab achieved platelets ≥30 × 10^9^/L and ≥2‐fold increase from baseline count confirmed on ≥2 occasions without bleeding (TP0003, *n* = 7/21; TP0006, *n* = 4/20), than the placebo groups (TP0003, *n* = 1/12; TP0006, *n* = 1/10).

In the OLE, 7/43 patients (16.3%) initially achieved stable platelets ≥50 × 10^9^/L. Two of these patients completed the study prior to local approval of the protocol amendments and received every 2‐week rozanolixizumab. Five switched to weekly dosing: two at Week 15, one at Week 25 and two at Week 40 (depending on local approval of protocol amendments). Figure S3 shows examples of patient‐level data that illustrate the further decrease of IgG associated with increased dosing frequency, and maintenance of platelet counts after switching from every 2‐week to weekly dosing.

### Safety

In the double‐blind studies, more TEAEs were reported with rozanolixizumab than with placebo (Table 2). Reported TEAEs were mostly mild or moderate. No apparent changes in safety profile were identified following the switch to weekly dosing in the OLE. The most frequently reported TEAEs were headache, pyrexia and nausea. No deaths were reported in any study. There were 15 events of COVID‐19 in 14 patients; two patients, in the OLE, required hospitalisation.

**TABLE 2:** Number of patients with treatment‐emergent adverse events.

	TP0003 (double‐blind)		TP0006 (double‐blind)		TP0004 (OLE)		
	RLZ	Placebo	RLZ	Placebo	Weekly^a^	Q2W^a^	Overall^b^
	*n* = 21	*n* = 12	*n* = 20	*n* = 10	*n* = 22	*n* = 42	*N* = 43
Any TEAE, *n* (%)	18 (85.7)	9 (75.0)	19 (95.0)	6 (60.0)	13 (59.1)	37 (88.1)	39 (90.7)
TEAEs leading to discontinuation of the study drug, *n* (%)	1 (4.8)	0	2 (10.0)	0	0	0	0
Treatment‐related TEAEs, *n* (%)	15 (71.4)	3 (25.0)	15 (75.0)	4 (40.0)	7 (31.8)	23 (54.8)	25 (58.1)
TEAEs leading to dose modification, *n* (%)	1 (4.8)	0	1 (5.0)	0	1 (4.5)	1 (2.4)	2 (4.7)
TEAEs CTCAE Grade 3 and above,^c^ *n* (%)	7 (33.3)	1 (8.3)	6 (30.0)	1 (10.0)	2 (9.1)	9 (21.4)	10 (23.3)
TEAEs resulting in temporary treatment discontinuation, *n* (%)	3 (14.3)	2 (16.7)	3 (15.0)	2 (20.0)	6 (27.3)	6 (14.3)	12 (27.9)
Most frequently reported non‐serious TEAEs (>10% patients in any treatment group), *n* (%)							
Headache	14 (66.7)	5 (41.7)	12 (60.0)	2 (20.0)	6 (27.3)	15 (35.7)	18 (41.9)
Pyrexia	9 (42.9)	0	6 (30.0)	0	2 (9.1)	7 (16.7)	9 (20.9)
Nausea	5 (23.8)	1 (8.3)	3 (15.0)	0	1 (4.5)	4 (9.5)	5 (11.6)
Vomiting	4 (19.0)	0	NA	NA	NA	NA	NA
Anaemia	3 (14.3)	2 (16.7)	2 (10.0)	0	NA	NA	NA
Diarrhoea	2 (9.5)	2 (16.7)	2 (10.0)	0	2 (9.1)	3 (7.1)	5 (11.6)
COVID‐19	1 (4.8)	2 (16.7)	1 (5.0)	1 (10.0)	4 (18.2)	3 (7.1)	7 (16.3)
Seasonal allergy	0	2 (16.7)	0	0	0	0	0
Serious TEAEs, *n* (%)^d^	2 (9.5)	1 (8.3)	5 (25.0)	1 (10.0)	2 (9.1)	7 (16.7)	9 (20.9)
Immune thrombocytopenia	1 (4.8)	0	0	1 (10.0)	1 (4.5)	0	1 (2.3)
Urethritis	1 (4.8)	0	0	0	0	0	0
Haemorrhage	0	1 (8.3)	0	0	0	1 (2.4)	1 (2.3)
Platelet count decreased	0	0	1 (5.0)	1 (10.0)	0	1 (2.4)	1 (2.3)
Vomiting	0	0	1 (5.0)	0	0	0	0
Pneumonia viral	0	0	1 (5.0)	0	0	0	0
Head injury	0	0	1 (5.0)	0	0	0	0
Limb injury	0	0	1 (5.0)	0	0	0	0
Skin injury	0	0	1 (5.0)	0	0	0	0
Headache	0	0	1 (5.0)	0	0	0	0
Dizziness	0	0	1 (5.0)	0	0	0	0
Urticaria	0	0	1 (5.0)	0	0	0	0
COVID‐19	0	0	0	0	0	2 (4.8)	2 (4.7)
Uterine haemorrhage	0	0	0	0	0	2 (4.8)	2 (4.7)
Joint dislocation	0	0	0	0	0	1 (2.4)	1 (2.3)
Radius fracture	0	0	0	0	0	1 (2.4)	1 (2.3)
Leiomyoma	0	0	0	0	0	1 (2.4)	1 (2.3)
Purpura	0	0	0	0	0	1 (2.4)	1 (2.3)
Skin haemorrhage	0	0	0	0	0	1 (2.4)	1 (2.3)
Pyrexia	0	0	0	0	1 (4.5)	0	1 (2.3)

Abbreviations: COVID‐19, coronavirus disease 2019; CTCAE, Common Terminology Criteria for Adverse Events; *n*, number of patients reporting the event; NA, not applicable (TEAE was not reported in >10% of patients in these studies); OLE, open‐label extension; Q2W, every 2 weeks; RLZ, rozanolixizumab; TEAE, treatment‐emergent adverse event.

^a^ Number of participants who received weekly or every 2‐week dosing at any time during the open‐label study.

^b^ *N* for all participants may be less than the sum of *N* for weekly and every 2‐week dosing, as patients may have experienced TEAEs on both weekly and every 2‐week dosing.

^c^ Or TEAEs rated as ‘severe’ for events with no CTCAE classification.

^d^ Number of patients who reported a serious TEAE; patients could have had >1 serious TEAE.

Across the three studies, five serious TEAEs in three patients, all receiving rozanolixizumab, were considered treatment‐related by the investigator: one event each of headache, vomiting and dizziness in one patient and urticaria in another patient (TP0006); and pyrexia (OLE). No treatment‐related serious TEAEs were reported in TP0003.

Across the double‐blind studies, seven rozanolixizumab‐treated patients reported nine AESMs related to rozanolixizumab. In TP0003, four patients experienced five events of headache and one patient experienced one event of diarrhoea; two events of headache in the same patient led to discontinuation. In TP0006, one patient experienced one event of headache, which led to discontinuation, and one patient experienced one event each of vomiting and abdominal pain. In the OLE, two patients, both on the every 2‐week regimen, had a total of two events of headache which were considered treatment‐related. No opportunistic infection or thromboembolic event was reported.

Except for pyrexia, no vital‐sign‐related or electrocardiogram‐related TEAEs were reported. Laboratory results generally remained within normal ranges in both groups; no abnormalities were considered clinically relevant changes from baseline.

### PKs, pharmacodynamics and immunogenicity

Rozanolixizumab concentration was below or close to the limit of quantification in predose samples for patients dosed every 2 weeks. Peak rozanolixizumab plasma concentrations were achieved 3 days after the first dose (≈15 mg/kg; Week 1): geometric mean (GeoMean) concentration 37.6 μg/mL (TP0003) and 40.1 μg/mL (TP0006). Samples taken 2–5 days postdose in Week 13 (GeoMean concentration 5.4 [TP0003] and 7.9 [TP0006] μg/mL) and Week 23 (GeoMean concentration 17.1 [TP0003] and 3.5 [TP0006] μg/mL), following the 10 mg/kg maintenance dose, were variable but similar based on the 95% CI around the GeoMean (Table S2), and were within the expected range.^8^ Similar to every 2‐week dosing, and as anticipated,^10^, ^13^ rozanolixizumab concentration was also below the limit of quantification in predose samples during weekly dosing.

**FIGURE 2:**
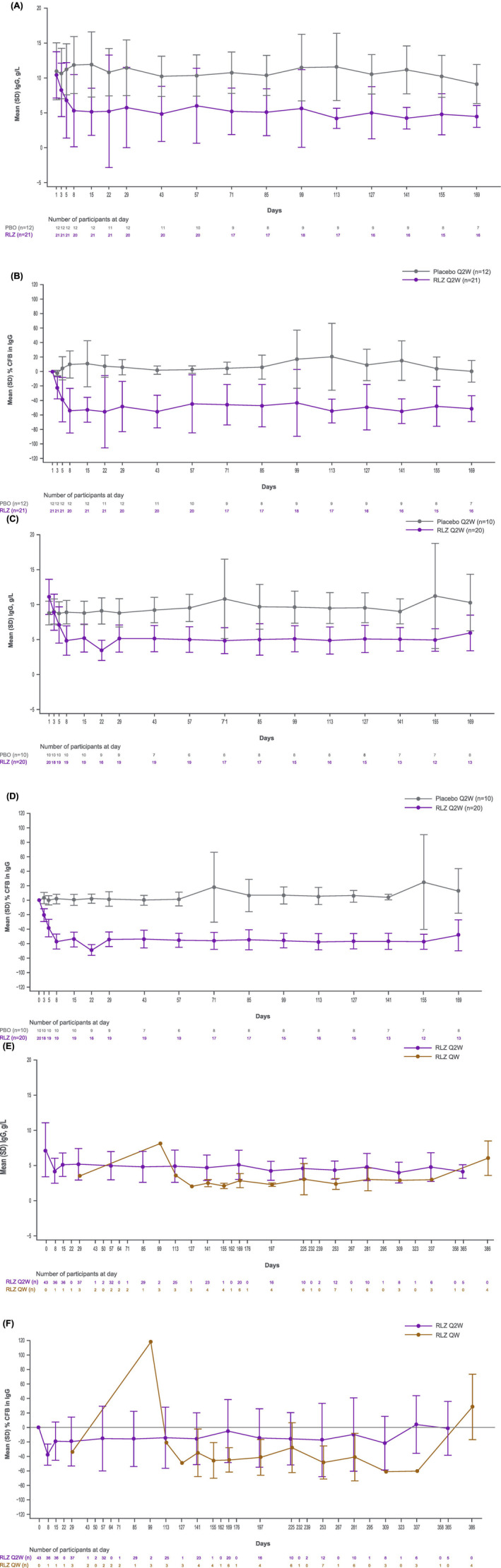
Mean serum and per cent change of IgG from baseline over time. (A) As observed (double‐blind; TP0003). (B) Change from baseline (double‐blind; TP0003). (C) As observed (double‐blind; TP0006). (D) Change from baseline (double‐blind; TP0006). (E) As observed (open‐label extension; TP0004)*. (F) Change from baseline^†^ (open‐label extension; TP0004)*. *Data points are only included where *n* = 3 or more. ^†^Baseline is open‐label extension baseline (approximately 5 g/L). CFB, change from baseline; IgG, immunoglobulin G; Q2W, every 2 weeks; QW, weekly; RLZ, rozanolixizumab.

In rozanolixizumab‐treated patients, a reduction from baseline (TP0003, 22%; TP0006, 20%) in mean total IgG was observed by Day 3 (Figure 2A–D). By Day 8, IgG levels had decreased further to 54% and 58% of baseline in TP0003 and TP0006, respectively. In the placebo groups, mean total IgG was stable during the double‐blind treatment periods. In the OLE, greater reductions from baseline were observed in total mean IgG concentrations with weekly dosing (2 g/L) compared to every 2‐week dosing (5 g/L) (Figure 2E,F). No clinically meaningful decreases in albumin levels were observed in any study; mean levels were within the normal range.

Variable ADA levels were observed throughout. At double‐blind study baseline, one patient was ADA‐positive (TP0006). Including all sampling points postbaseline, 15/21 (TP0003) and 12/16 (TP0006) patients had ≥1 positive sample for treatment‐emergent ADAs. Post‐OLE baseline, 26/43 patients had treatment‐emergent ADAs at any point. A total of 3/20 and 5/16 patients had persistent ADA positivity in TP0003 and TP0006 respectively. In the OLE, 11/24 patients and 4/17 patients randomised to placebo, respectively, in the double‐blind studies showed persistent ADA positivity.

## DISCUSSION

These phase 3, randomised, placebo‐controlled, double‐blind studies and their OLE demonstrated that rozanolixizumab was well tolerated and induced clinically meaningful increases in platelet counts in most patients within 1 week of administration in this predominantly chronic ITP population. These findings are consistent with those from the rozanolixizumab phase 2 study in a similar patient population.^9^

However, with every 2‐week dosing in the present studies, and despite sustained IgG reduction, platelet count increases following each infusion were not maintained over the dosing interval. Thus, total IgG decreases of approximately 50% were not associated with sustained platelet count elevations, whereas weekly dosing resulted in greater IgG decreases. The rapid increase in platelet counts observed in the phase 2 study^9^ and the present studies, both after the starting dose and following each subsequent dose, is not consistent with the time course of the IgG level decrease. Hypothesis testing using a quantitative system pharmacology model (not shown) predicted that every 2‐week dosing would not sustain an FcRn receptor occupancy level above 50% throughout the dosing interval, while weekly dosing would.

The rapid onset of action of rozanolixizumab, evident by data on Day 8, was demonstrated with both the starting dose of ≈15 and ≈10 mg/kg during repeated dosing in the double‐blind studies, and in most patients (10/17) who started the OLE study on ≈10 mg/kg rozanolixizumab after having been on placebo. Mean platelet counts in patients who switched to weekly dosing in the OLE were ≥50 × 10^9^/L at all visits after Week 26, suggesting that weekly dosing of rozanolixizumab may result in sustained, increased platelet counts.

A lesser response among rozanolixizumab‐treated patients was observed in one double‐blind study (TP0006) compared to the other (TP0003). Patients in TP0006 had a median ITP duration of approximately 12 years, compared with approximately 5 years in TP0003; there was also a higher Asian‐to‐European patient ratio and a higher median number of prior ITP medications used in TP0006 versus TP0003, all of which may have contributed to lower response rates.

The observed rapid clearance of rozanolixizumab was anticipated based on its known PK characteristics, and is similar to data from animal models, healthy volunteers and patients with myasthenia gravis receiving weekly rozanolixizumab. Furthermore, upon binding to FcRn, rozanolixizumab inhibits its own recycling system, thus increasing its clearance.^10^, ^13^, ^14^ Sustained IgG reduction throughout the study, following the rapid reduction in IgG levels from baseline after the first dose, may reduce the level of ADAs. IgG levels were reduced further with weekly than every 2‐week dosing in the OLE, again supporting a more pronounced pharmacodynamic effect of ongoing treatment, consistent with findings from other studies.^7^, ^10^

Safety data from these studies were consistent with the known safety profile of rozanolixizumab.^9^, ^10^, ^14^ The level of reduction in IgG observed with rozanolixizumab treatment did not predispose patients to infections, with similar rates in both rozanolixizumab and placebo groups. This is consistent with many other FcRn inhibition studies for a variety of indications using several different preparations.^10^, ^15^, ^16^ No apparent changes in the safety profile were identified following a switch to weekly dosing. While more TEAEs were observed with every 2‐week dosing than with weekly dosing, patients spent less time overall on the weekly dosing regimen.

Efgartigimod, a human IgG1 Fc fragment FcRn antagonist, has demonstrated clinically relevant increases in platelet count with associated reductions in IgG levels in a phase 3 study of patients with primary ITP following intravenous administration (ADVANCE IV).^16^ However, a second phase 3 study using the SC efgartigimod and hyaluronidase‐qvfc (VYVGART Hytrulo®) formulation in 207 patients with chronic/persistent primary ITP (ADVANCE‐SC) failed to confirm these increases.^17^ Overall, these sets of findings also demonstrate a disconnect between the pharmacodynamic effect of IgG reduction and that of increase of platelet numbers. In the present studies, while the initial increase in platelet count was rapid and in line with IgG reduction, platelet numbers progressively decreased beyond Day 8 prior to the next dose of rozanolixizumab. The rapid initial rise in platelet numbers could be attributed to inhibition of macrophage phagocytosis rather than by reduction of autoantibody titres.^18^, ^19^ As for other IgG‐mediated diseases,^10^ it is presumed that IgG autoantibody levels in ITP decrease in parallel to the overall serum IgG concentration, but this has not been demonstrated.

Administration of rozanolixizumab every 2 weeks, rather than weekly, likely contributed to the minimal difference in efficacy end‐points between rozanolixizumab and placebo. Due to the early study termination, no formal statistical analyses of efficacy end‐points were conducted, and no statistical comparison of weekly to every 2‐week dosing could be done, so all data are summarised descriptively. Further, due to the relatively small sample size compared to that planned, interpretation of the data should be made with caution. Nonetheless, weekly dosing appeared to have a better effect on platelet counts compared to every 2‐week dosing.

In conclusion, the results from these studies reflect the known safety profile of rozanolixizumab, while also providing evidence of maintained clinically meaningful platelet count increases and reduced IgG levels with weekly dosing in patients with predominantly chronic ITP. Weekly administration of rozanolixizumab in a larger ITP patient population may show sustained clinical benefits.

## AUTHOR CONTRIBUTIONS

NC, SC, RLG, BH, AL, PS and RS contributed to the concept or study design. NC, JBB, MK, YM and DJK contributed to the acquisition of data by enrolling patients. SC contributed to the statistical analysis. All authors contributed to the interpretation of the data. All authors had full access to study data, reviewed, edited and provided final approval of the manuscript content, and had final responsibility for the decision to submit for publication.

## CONFLICT OF INTEREST STATEMENT

Nichola Cooper has received consultancy fees from Amgen, argenx, Novartis, Principia, Rigel, Sanofi, and UCB, and research support from Novartis and Rigel. James B. Bussel has received consultancy fees from argenx, Sobi, Momenta (now Johnson and Johnson), Novartis, Sanofi, Platelet Disorders Support Association and UCB and has served on a Data and Safety Monitoring Board for UCB. Maciej Kaźmierczak has nothing to disclose. Yoshitaka Miyakawa has received consultancy fees from argenx, Kissei and Zenyaku, and research support from Kissei, Novartis, Sanofi, and UCB. Sarah Cluck, Rocío Lledó García, Birgit Haier, Andreea Lavrov and Rose Snipes are employees and shareholders of UCB. Puneet Singh is an employee of UCB and a shareholder of UCB and GSK. David J. Kuter has received consultancy fees and research funding from Actelion (Syntimmune), Alnylam, Amgen, argenx, BioCryst, BMS, Immunovant, Principia, Rigel, Takeda (Bioverativ) and UCB; consultancy fees from Caremark, Cellphire, Cellularity, CRICO, Daiichi Sankyo, Dova, Genzyme, Hengrui, Incyte, Merck Sharp & Dohme, Momenta, Novartis, Pfizer, Platelet BioGenesis, Platelet Disorder Support Association, Sanofi, Shionogi, Shire, UpToDate and Alpine.

## ETHICS STATEMENT

The studies were conducted under the auspices of Institutional Review Boards or Independent Ethics Committees, as defined in local regulations, International Council for Harmonization‐Good Clinical Practice, and in accordance with the ethical principles that have their origin in the Declaration of Helsinki.

## PATIENT CONSENT STATEMENT

All patients provided written informed consent.

## CLINICAL TRIAL REGISTRATION

These studies are registered at ClinicalTrials.gov. TP0003: NCT04200456; registered at https://clinicaltrials.gov/study/NCT04200456. TP0006: NCT04224688; registered at https://clinicaltrials.gov/study/NCT04224688. TP0004: NCT04596995; registered at https://clinicaltrials.gov/study/NCT04596995.

## Supporting information


Data S1.

## Data Availability

Data from this trial may be requested by qualified researchers 6 months after product approval in the US and/or Europe, or after global development is discontinued, and 18 months after trial completion. Investigators may request access to anonymised individual patient‐level data and redacted trial documents, which may include analysis‐ready datasets, study protocol, annotated case report form, statistical analysis plan, dataset specifications and clinical study report. Prior to the use of the data, proposals need to be approved by an independent review panel at www.vivli.org and a data‐sharing agreement will need to be signed. All documents are available in English only, for a prespecified time, typically 12 months, on a password‐protected portal. This plan may change if the risk of re‐identifying trial participants is determined to be too high after the trial is completed; in this case, and to protect participants, individual patient‐level data would not be made available.
