# Supplementary material for: Inhibition of FcRn with rozanolixizumab in adults with immune thrombocytopenia: Two randomised, double‐blind, placebo‐controlled phase 3 studies and their open‐label extension
Source: Br J Haematol. 2024 Nov 18;206(2):675–88. doi: 10.1111/bjh.19858 (PMC11829145; doi:10.1111/bjh.19858)
Supplement: Supplementary file 1 — Data S1. [file BJH-206-675-s001.docx]

**Inhibition of FcRn with rozanolixizumab in adults with immune thrombocytopenia (ITP): Two randomized, double-blind, placebo-controlled, Phase 3 studies and their open-label extension**

Nichola Cooper^1^, James Bussel^2^, Maciej Kaźmierczak^3^, Yoshitaka Miyakawa^4^, Sarah Cluck^5^, Rocío Lledó García^5^, Birgit Haier^6^, Andreea Lavrov^6^, Puneet Singh^5^, Rose Snipes^7^, David J. Kuter^8^

^1^Imperial College London, London, UK; ^2^Weill Cornell Medicine – New York Presbyterian Hospital, New York, NY, USA; ^3^Poznań University of Medical Sciences, Poznań, Poland; ^4^Saitama Medical University, Saitama, Japan; ^5^UCB, Slough, UK; ^6^UCB, Monheim, Germany; ^7^UCB, Morrisville, NC, USA; ^8^Hematology Division, Massachusetts General Hospital, Boston, MA, USA

**Corresponding author:** Dr Nichola Cooper
Email: [n.cooper@imperial.ac.uk](mailto:n.cooper@imperial.ac.uk)

**Supplementary Material**

**Supplementary Figure 1. Study design**

A. TP0003 and TP0006 (double-blind)


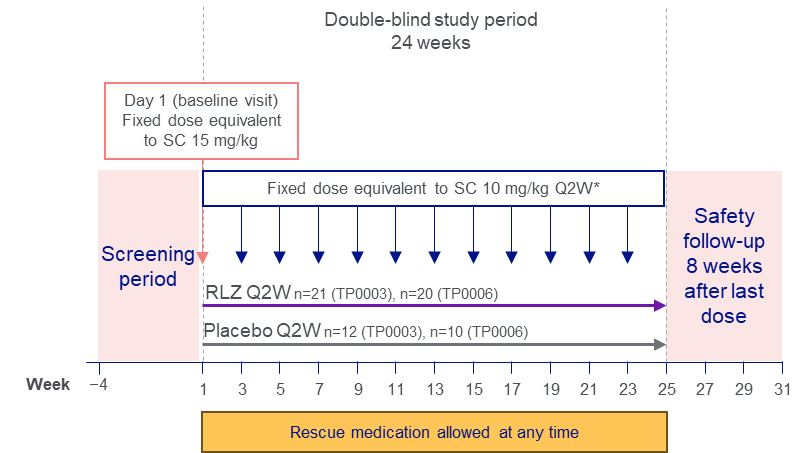


B. TP0004 (open-label extension)


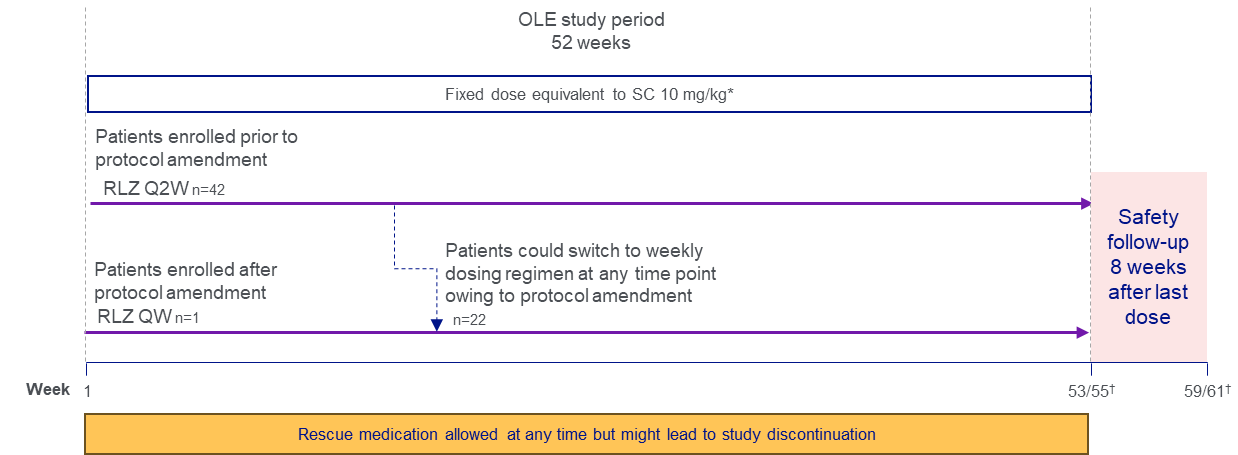


*Up- or down-titration if required (Supplementary Table 1). ^†^Week number differed depending on the dosing regimen: QW dosing, Weeks 53 and 59; Q2W dosing, Weeks 55 and 61.

OLE, open-label extension; Q2W, every 2 weeks; QW, weekly; RLZ, rozanolixizumab; SC subcutaneous.

**Supplementary Figure 2. Patient disposition**

A. TP0003 (double-blind)


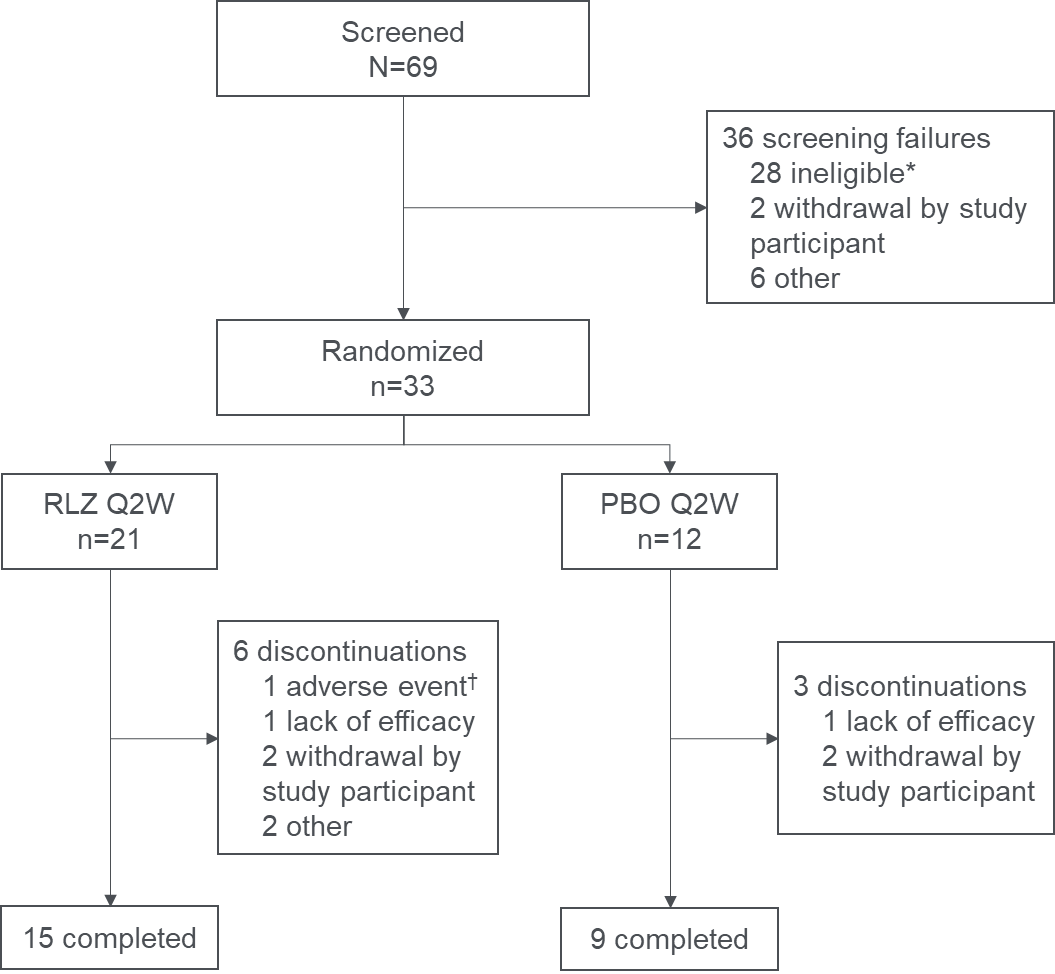


*Inclusion criteria not met: platelet count measurement at screening and at baseline (Day 1) with an average of the two <30×10^9^/L and no single count may be >35×10^9^/L (using local laboratories) (n=9); considered reliable and capable of adhering to the protocol, visit schedule or medication intake according to the judgment of the investigator (n=2); capable of giving signed informed consent which includes compliance with the requirements and restrictions listed in the Informed Consent Form (n=1). Exclusion criteria met: known TB infection, at high risk of acquiring TB infection, or latent tuberculosis infection, or current/history of nontuberculous mycobacterial infection (n=6); glycosylated hemoglobin value >8% at the Screening Visit (n=3); splenectomized participant without adequate vaccination against *S. pneumoniae*, *N. meningitidis*, and *H. influenzae* (n=3); current or medical history of IgA deficiency, or a measurement of IgA <50mg/dL at the Screening Visit (n=2); absolute neutrophil count <1500 cells/mm^3^ (n=1); treated with prohibited immunosuppressants, biologics and other therapies within a timeframe shorter than permitted (n=1); history of arterial or venous thromboembolism (e.g., stroke, transient ischemic attack, myocardial infarction, deep vein thrombosis or pulmonary embolism) within the 6 months prior to randomization or requires anticoagulant treatment (n=1); not considered capable of adhering to the protocol visit schedule or medication intake according to the judgment of the investigator (n=1). One patient had no reason listed. Patients could have more than one reason for ineligibility.

^†^The adverse event leading to discontinuation was one event of treatment-related severe headache.

PBO, placebo; Q2W, every 2 weeks; RLZ, rozanolixizumab; TB, tuberculosis.

B. TP0006 (double-blind)


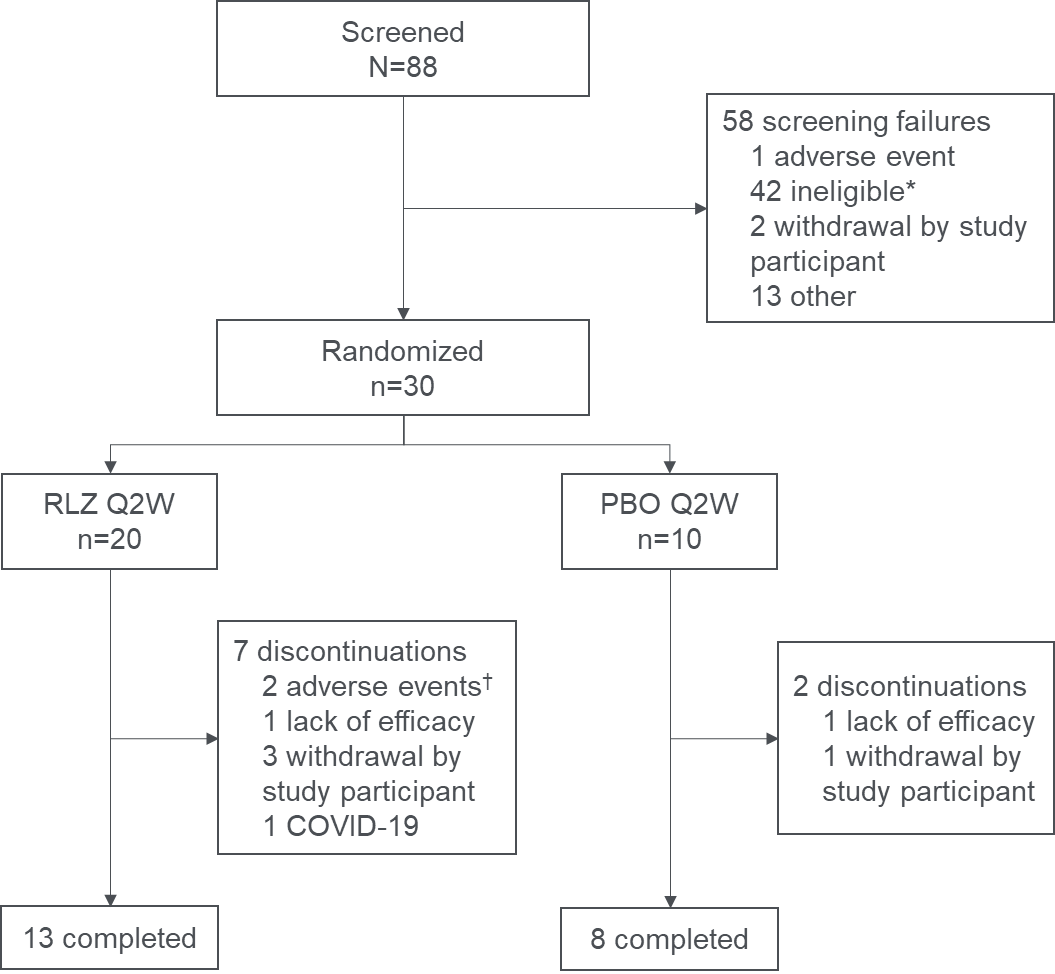


*Inclusion criteria not met: platelet count measurement at screening and at baseline (Day 1) with an average of the two <30×10^9^/L and no single count may be >35×10^9^/L (using local laboratories) (n=14); documented history of low platelet count (<30×10^9^/L) prior to screening (n=5); if taking allowed drugs, must be on stable doses during defined time periods prior to baseline (Day 1) (n=2); documented intolerance or insufficient response to two or more appropriate standard-of-care ITP medications (including but not limited to corticosteroids, immunoglobulins, TPO-RAs, azathioprine, danazol, cyclophosphamide, and/or rituximab or other immunosuppressants) prior to screening (n=2); IgG level of >5.5 g/L if non-splenectomized or ≥6.5 g/L if splenectomized at screening (n=1); capable of giving signed informed consent which includes compliance with the requirements and restrictions listed in the Informed Consent Form (n=1). Exclusion criteria met: known TB infection, at high risk of acquiring TB infection, or latent TB infection, or current/history of nontuberculous mycobacterial infection (n=7); glycosylated hemoglobin value >8% at the Screening Visit (n=3); not considered capable of adhering to the protocol visit schedule, or medication intake according to the judgment of the investigator (n=2); partial thromboplastin time ≥1.5× upper limit of normal or International Normalized Ratio ≥1.5 at the screening Visit (n=1); current or medical history of IgA deficiency, or a measurement of IgA <50 mg/dL at the screening visit (n=1); positive for HIV at the Screening Visit (n=1); received any biological agent, other than specified as permitted, in the past 3 months or within 5 half-lives prior to baseline (whichever was longer) (n=1); 12-lead ECG with changes considered to be clinically significant upon medical review (n=1); treated with prohibited immunosuppressants, biologics and other therapies within a timeframe shorter than permitted (n=1); previously received rozanolixizumab in another clinical study (n=1); bilirubin >1.5 × ULN (unless confirmed Gilbert’s syndrome) or elevations only in total bilirubin, fractionate bilirubin to identify possible undiagnosed Gilbert’s syndrome (i.e., direct bilirubin <35%) at the Screening Visit (n=1). Patients could have more than one reason for ineligibility.

^†^The adverse events leading to discontinuation were one event of treatment-related serious headache and one event of treatment-related severe urticaria.

COVID-19, coronavirus disease 2019; ECG, electrocardiogram; ITP, immune thrombocytopenia; PBO, placebo; Q2W, every 2 weeks; RLZ, rozanolixizumab; TB, tuberculosis; TPO-RA, thrombopoietin-receptor agonist.

C. TP0004 (open-label extension)


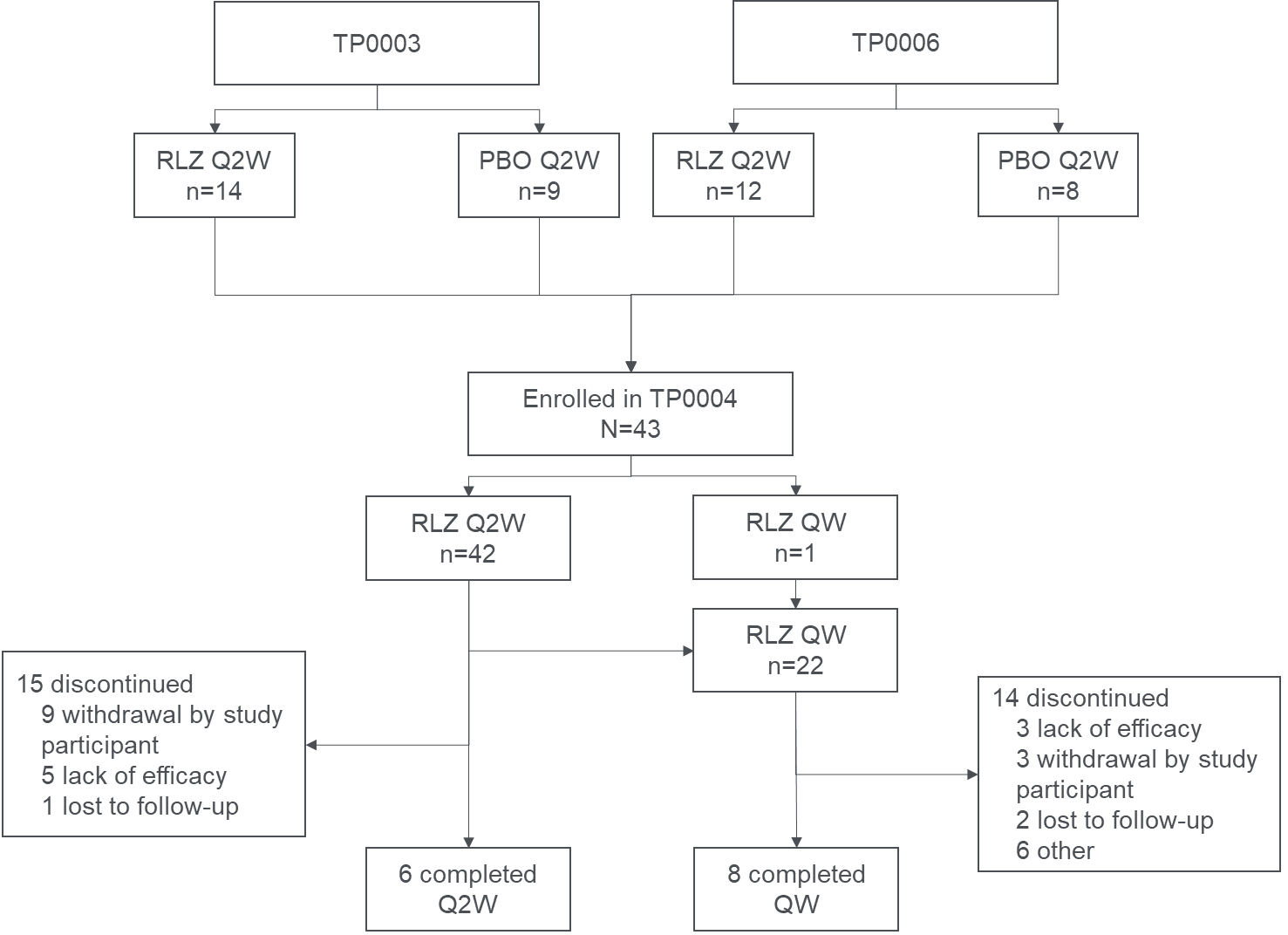


COVID-19, coronavirus disease 2019; PBO, placebo; Q2W, every 2 weeks; QW, weekly; RLZ, rozanolixizumab.

**Supplementary Figure 3. Individual platelet counts and IgG concentrations over time in patients who switched from every-2-week to weekly dosing**

1. Patient 1 (previously receiving rozanolixizumab in the double-blind studies)


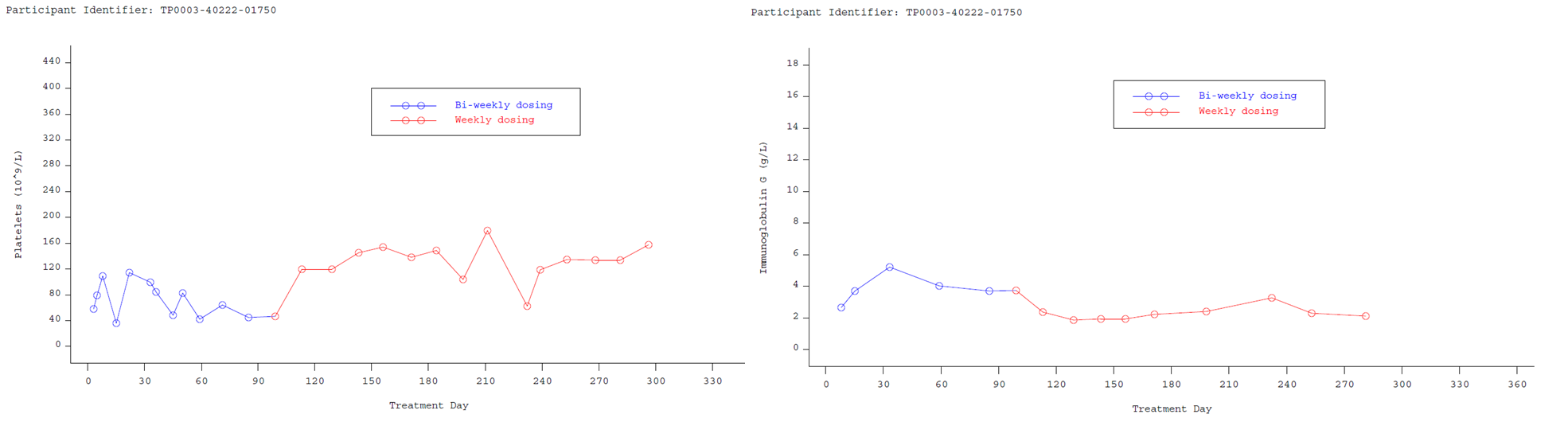


1. Patient 2 (previously receiving placebo in the double-blind studies)


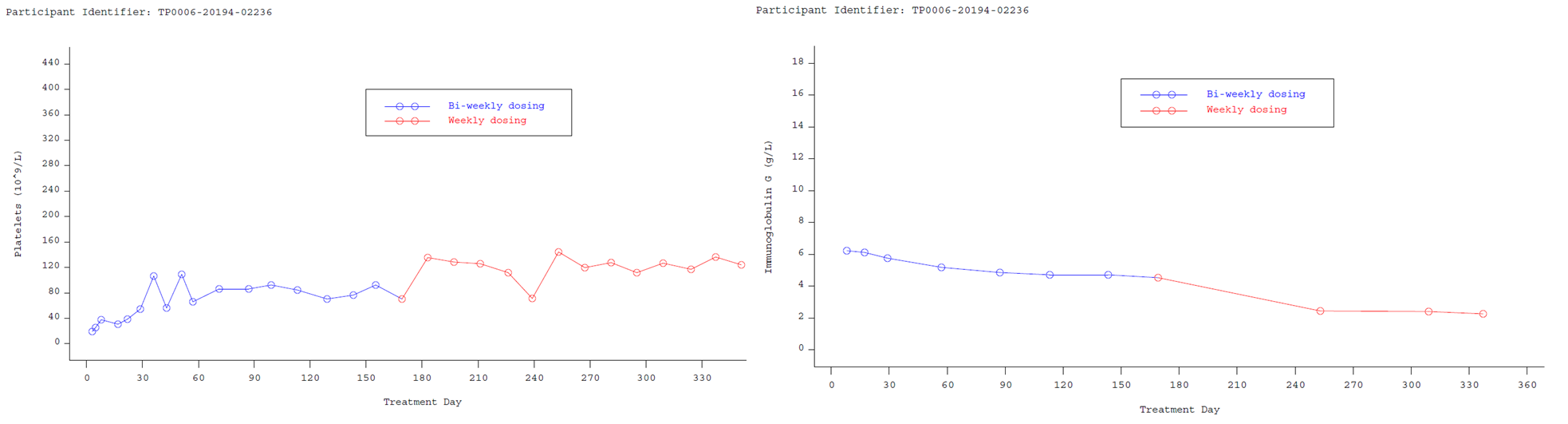


**Supplementary Table 1. Rozanolixizumab dose levels and adjustments**

A. Rozanolixizumab dose levels and weight tiers

|  | **Dose equivalent** | | | |
| --- | --- | --- | --- | --- |
| **Bodyweight** | **15 mg/kg  starting dose** | **10 mg/kg  maintenance dose level 1** | **7 mg/kg maintenance dose level 2** | **~4 mg/kg maintenance dose level 3** |
| >35 to <50 kg | 560 mg | 420 mg | 280 mg | 280 mg  No weight adjustment |
| ≥50 to <70 kg | 840 mg | 560 mg | 420 mg |  |
| ≥70 to <100 kg | 1120 mg | 840 mg | 560 mg |  |
| ≥100 kg | 1680 mg | 1120 mg | 840 mg |  |

B. Rozanolixizumab dose adjustments for every-2-week dosing

| **Platelet count result*** | **Dose adjustment (based on weekly platelet counts)** |
| --- | --- |
| <50×10^9^/L (on at least two consecutive visits) | Increase by one dose level.  If patient is on 10 mg/kg equivalent dose, this dose may be continued although platelet count is <50×10^9^/L, provided platelets are ≥30×10^9^/L and rescue therapy is not deemed necessary |
| ≥50×10^9^/L to ≤200×10^9^/L^†^ | Continue with current dose level |
| >200×10^9^/L to <400×10^9^/L (on at least two consecutive visits) | Decrease by one dose level, unless study participant is on maintenance dose of ~4 mg/kg |
| ≥400×10^9^/L | Stop rozanolixizumab treatment. Once the platelet count is ≤200×10^9^/L, but ≥50×10^9^/L, reinitiate treatment decreased by one dose level |

C. Rozanolixizumab dose adjustments for weekly dosing

| **Platelet count result*** | **Dose adjustment (based on weekly platelet counts)** |
| --- | --- |
| <10×10^9^/L | Increase by one dose level, unless study participant is on maintenance dose of 10 mg/kg. Additional rescue therapy is highly recommended |
| ≥10×10^9^/L to <30×10^9^/L (on at least two consecutive visits) | Increase by one dose level, unless study participant is on maintenance dose of 10 mg/kg. Additional rescue therapy is recommended |
| ≥30×10^9^/L to <50×10^9^/L (on at least two consecutive visits) | Increase by one dose level, unless study participant is on maintenance dose of 10 mg/kg |
| ≥50×10^9^/L to ≤150×10^9^/L^†^ | Continue with current dose level |
| >150×10^9^/L to <400×10^9^/L (on at least two consecutive visits) | Decrease by one dose level, unless study participant is on maintenance dose of ~4 mg/kg |
| ≥400×10^9^/L | Stop IMP treatment. Once the platelet count is ≤150×10^9^/L, reinitiate treatment decreased by one dose level |

*All analyses of platelet counts were based on local laboratory results. ^†^Due to the interindividual variable platelet response in some study participants, platelet count may abruptly fall below 50×10^9^/L after dose reduction or treatment discontinuation. In these cases, if clinically appropriate higher cut-off levels of platelet count for dose reduction (>150×10^9^/L) may be considered according to medical judgement, but should not exceed 250×10^9^/L. IMP, investigational medicinal product.

**Supplementary Table 2. Rozanolixizumab plasma concentrations geometric mean (μg/mL), every-2-week dosing**

|  | **TP0003 (double-blind)** | | | | **TP0006 (double-blind)** | | | |
| --- | --- | --- | --- | --- | --- | --- | --- | --- |
|  | Dose | n | GeoMean | 95% CI | Dose | n | GeoMean | 95% CI |
| Week 1, Day 3 post dose | 15 mg/kg | 21 | 37.6 | 19.1, 74.1 | 15 mg/kg | 17 | 40.1 | 28.5, 56.5 |
| Week 13, Day 2‒5 post dose | 10 mg/kg | 9 | 5.4 | 0.8, 37.1 | 10 mg/kg | 10 | 7.9 | 2.9, 21.7 |
| Week 23, Day 2‒5 post dose | 10 mg/kg | 6 | 17.1 | 9.7, 30.4 | 10 mg/kg | 7 | 3.5 | 0.3, 45.6 |
|  | **TP0004 (OLE)** | | | |  |  |  |  |
|  | Dose | n | GeoMean | 95% CI |  |  |  |  |
| Week 1, Day 3 post dose | 7 mg/kg | 4 | 6.1 | 0.5, 79.9 |  |  |  |  |
|  | 10 mg/kg | 35 | 13.3 | 7.2, 24.4 |  |  |  |  |
| Week 5, Day 2–5 post dose | 7 mg/kg | 1 | – | – |  |  |  |  |
|  | 10 mg/kg | 15 | 5.7 | 2.0, 16.2 |  |  |  |  |
| Week 13, Day 2‒5 post dose | 7 mg/kg | 1 | – | – |  |  |  |  |
|  | 10 mg/kg | 14 | 9.9 | 3.7, 26.6 |  |  |  |  |
| Week 21, Day 2–5 post dose | 7 mg/kg | 1 | – | – |  |  |  |  |
|  | 10 mg/kg | 12 | 8.4 | 2.0, 35.7 |  |  |  |  |
| Week 41, Day 2–5 post dose | 7 mg/kg | 0 | – | – |  |  |  |  |
|  | 10 mg/kg | 4 | 12.7 | 2.5, 64.8 |  |  |  |  |

CI, confidence interval; GeoMean, geometric mean; OLE, open-label extension.
